# Job Satisfaction Among Frontline Caregivers: The Mediating Role of Psychological Safety and Personality Traits †

**DOI:** 10.3390/healthcare14091208

**Published:** 2026-04-30

**Authors:** Xinyi Min, Haru Kaneko, Takeshi Takahama, Yuka Shono, Yuji Tanaka, Sozo Inoue

**Affiliations:** 1 Sozo Lab, Department of Life Science and Systems Engineering, Kyushu Institute of Technology, 2-4 Hibikino, Wakamatsu-ku, Kitakyushu-shi, Fukuoka 808-0196, Japan; 2 TRYT Inc., Art Village Osaki Central Tower 17F, 1-2-2 Osaki, Shinagawa-ku, Tokyo 141-0032, Japan

**Keywords:** care facility, caregiver, psychological safety, personality, job satisfaction

## Abstract

**Background**: Job satisfaction remains a persistent concern in care facilities, where frontline caregivers work in highly relational and emotionally demanding environments. Although both personality traits and psychological safety have been associated with job satisfaction, their relative contributions and potential interrelationships in routine care settings remain unclear. **Methods**: Drawing on organizational and psychological theory, we hypothesized a priori that personality traits, psychological safety, and job satisfaction would be statistically associated, with psychological safety mediating the relationship between personality traits and job satisfaction. A cross-sectional questionnaire survey was conducted in care facilities, and the final analytical sample consisted of 183 frontline caregivers selected according to predefined inclusion criteria. Structural equation modeling (SEM) was used to test the hypothesized mediation model. Group comparison analyses examined differences in psychological safety across levels of job satisfaction. In addition, machine learning models were applied to explore predictive patterns among personality traits, psychological safety, and job satisfaction. **Results**: In the SEM analyses, psychological safety was significantly associated with job satisfaction (β = 0.207, *p* < 0.05), although the effect size was modest. Personality traits did not show direct associations with job satisfaction. Instead, agreeableness (β = 0.232, *p* < 0.01) and neuroticism (β = −0.235, *p* < 0.01) were associated with psychological safety, which, in turn, was related to job satisfaction. Bootstrap resampling supported the presence of significant indirect associations. At the item level, communication-related aspects of psychological safety were particularly salient. Caregivers with different levels of job satisfaction differed most clearly in communication-related items. Consistent with this pattern, exploratory machine learning analyses (XGBoost) also identified psychological safety as a more important predictor of job satisfaction than personality traits. **Conclusions**: These findings suggest that, among frontline caregivers working in healthcare facilities, psychological safety was more closely associated with job satisfaction than individual personality traits. In particular, the ability to speak openly and raise concerns was more strongly associated with higher levels of job satisfaction within this care context. Personality traits were associated with job satisfaction primarily through their relationship with psychological safety rather than through direct associations.

## 1. Introduction

At present, turnover rates in the healthcare and welfare sectors remain persistently high. According to the Ministry of Health, Labour and Welfare’s 2024 Employment Trend Survey, the number of workers who left employment in the “medical and welfare” sector reached 1.135 million, making it the second largest industry nationwide in terms of turnover, following the “wholesale and retail” sector [1]. Trends over the past several years show no substantial improvement in the number of employees leaving the sector.

Studies that examine the intention to leave of nursing professionals have consistently reported that job satisfaction is one of the strongest predictors of intention to leave [2]. Moreover, recent evidence has demonstrated that workforce instability has serious negative effects on patient outcomes, being associated with increases in medical errors, higher infection rates, delayed postoperative recovery, and even higher mortality rates [3,4]. Therefore, improving employees’ job satisfaction constitutes an urgent challenge. Prior research in healthcare settings has shown that job satisfaction is shaped by multiple organizational and interpersonal factors, including workload, managerial support, work environment, and team relationships [5]. Although much of this evidence originates from nursing research, caregiving work in long-term care facilities similarly involves intensive interpersonal interaction and teamwork, suggesting that workplace climate may also play an important role in shaping caregivers’ job satisfaction. One important aspect of workplace climate that has attracted increasing attention in recent years is psychological safety.

Psychological safety is a concept proposed by Amy C. Edmondson and refers to a state in which individuals can freely express opinions and questions without fear of criticism or rejection [6,7]. In healthcare settings, high levels of psychological safety are known to contribute to effective team communication, collaborative behavior, and improved patient safety. More broadly, psychological safety has been associated with a range of positive workplace outcomes, including learning behavior, work engagement, and job attitudes such as job satisfaction [8]. In contrast, workplaces with low psychological safety have been reported to experience increased anxiety and fear among caregivers, leading to frequent communication errors [9].

In addition, prior research has indicated that personality traits are associated with psychological responses, stress tolerance, and interpersonal behavior in the workplace. Moreover, Newman et al. (2017) [10] highlighted the need to adopt person–situation perspectives, such as trait activation theory, to better understand how psychological safety is linked to work-related outcomes. In particular, neuroticism has been associated with increased stress and burnout, whereas agreeableness and extraversion have been shown to promote interpersonal collaboration and teamwork [11,12]. These findings suggest that personality traits may relate to job satisfaction both directly and indirectly through workplace perceptions such as psychological safety.

However, relatively few studies have simultaneously examined personality traits and psychological safety within the same analytical framework among frontline caregivers in care facilities. The present study addresses this gap by examining the associations among personality traits, psychological safety, and job satisfaction in a sample of frontline caregivers. Based on existing literature, we expect that personality traits, psychological safety, and job satisfaction are statistically associated. Furthermore, we hypothesize a priori that psychological safety may function as a statistical mediator linking personality traits to job satisfaction.

Elements of this research have been presented in a previous publication [13]. In this article, we expand upon that work by adding relevant literature, strengthening comparisons with prior studies, and deepening the discussion of related research. This study aims to analyze the key factors influencing the job satisfaction of frontline caregivers. The characteristics examined encompass intrinsic factors such as personality traits, external factors like psychological safety, and objective factors including years of service and institutional type. Of the 521 responses collected nationwide from care facilities, 183 individuals directly engaged in caregiving were selected for analysis, and factors affecting job satisfaction were examined from multiple angles.

Accordingly, the present study addresses the following research questions:

- Research Question 1 (RQ1): What role do personality traits and psychological safety play in explaining job satisfaction among frontline caregivers in care facilities?
- Research Question 2 (RQ2): Are personality traits directly associated with job satisfaction, or are their associations statistically mediated through psychological safety in care facilities?
- Research Question 3 (RQ3): Which components of psychological safety are most strongly associated with differences in job satisfaction among frontline caregivers?

To address these research questions, structural equation modeling (SEM) is employed as a confirmatory framework to test the hypothesized mediation structure. Group comparison analyses are used to examine differences across job satisfaction levels, and machine learning approaches are applied as complementary methods to explore the relative contribution of psychological safety and personality traits.

## 2. Related Work

This section reviews prior research on personality, psychological safety, and job satisfaction. These three perspectives together provide the conceptual foundation for the present study and clarify both the challenges in understanding job satisfaction in care settings and the specific focus of this research. These studies provide the foundation for the current research.

### 2.1. Research on Personality

Personality has been widely shown to strongly influence stress responses, interpersonal relationships, and collaborative behaviors in the workplace. In particular, neuroticism, agreeableness, and extraversion are closely related to psychological adaptation and interpersonal functioning. Bagdi et al. [14] focused on the interaction between emotional labor and personality, demonstrating the process by which psychosocial factors increase emotional exhaustion, a core component of burnout. These findings suggest that personality traits such as neuroticism and agreeableness influence workplace attitudes through stress tolerance and interpersonal behavior, supporting the necessity of incorporating personality into models of job satisfaction.

However, much of the existing research has mainly examined how personality relates to individual stress outcomes, such as emotional exhaustion or burnout. Fewer studies have explored how personality operates within team environments and how it may shape broader work attitudes through shared psychosocial climates. In particular, the potential mediating role of psychological safety in linking personality traits to job satisfaction remains underexamined.

### 2.2. Psychological Safety and the Organizational Environment

Psychological safety refers to a state in which individuals can speak up without fear of criticism or rejection and is largely shaped by organizational culture and the team environment. Prior research in healthcare and caregiving has shown that psychological safety is closely associated with the quality of the work environment, including leadership behaviors, openness of communication, a culture that tolerates errors, and team learning processes.

In workplaces with high psychological safety, staff can freely exchange opinions, information sharing and collaborative behaviors are facilitated, and psychological burden is consequently reduced. At the same time, research has shown that it can also promote business performance and achieve better work results [15]. Conversely, low psychological safety has been reported to lead to suppression of voice and stagnation of team learning, increasing anxiety and withdrawal, and negatively affecting employees’ work attitudes [16,17]. These findings indicate that psychological safety is a critical factor that directly influences attitude formation by mediating organizational environments. In care settings as well, higher psychological safety is likely to contribute to improved job satisfaction. Accordingly, psychological safety, alongside personality, is positioned as a major external determinant of job satisfaction.

However, most of the existing research has mainly examined psychological safety as a direct predictor of outcomes such as performance or work attitudes. Less attention has been paid to how psychological safety interacts with individual personality traits within the same model. In particular, few studies have empirically tested whether psychological safety functions as a bridge linking personality traits to job satisfaction. This gap is especially noticeable in long-term care settings, where frontline caregivers work in highly relational and communication-dependent environments. The present study aims to address this issue by examining personality traits and psychological safety together within an analytical framework.

### 2.3. Job Satisfaction

Prior studies on job satisfaction have reported that multiple workplace factors—such as heavy workload, emotional demands, lack of organizational support, and interpersonal friction—contribute to declines in satisfaction [12]. It has also been noted that collaborative organizational cultures and leadership behaviors shape employees’ work orientations and commitment, influencing the interaction between depletion of psychological resources and job satisfaction [2].

Consistent with this perspective, a recent systematic review of the pharmacy workforce synthesized evidence from over 80 studies and identified several particularly salient determinants of job satisfaction [18]. Burnout, stress, and workload emerged as the most frequently reported determinants, followed by work conditions and role clarity, professional development opportunities, earnings and benefits, and leadership support across diverse healthcare settings. Although conducted in the pharmacy context, these findings highlight organizational and psychosocial factors that are similarly relevant to other frontline caregiving roles. Together, this body of evidence reinforces the multifactorial and context-dependent nature of job satisfaction in healthcare settings.

At the same time, most studies have focused on listing important determinants rather than examining how individual traits and team environments work together. In particular, the potential role of psychological safety as a link between personality traits and job satisfaction has rarely been tested directly. By bringing these elements together within one model, this study aims to offer a more integrated perspective on job satisfaction in frontline caregiving settings.

### 2.4. SEM

Recent studies increasingly apply structural equation modeling to investigate complex pathways between psychological constructs and work outcomes. For example, Ring & Hult [19] used SEM to model how psychological safety and psychological contract breach impact burnout and work engagement among nurses.

Bennouna et al. [20] demonstrated a moderated mediation model in healthcare where psychological safety and job satisfaction mediated the impact of leader-member exchange on safety behaviors, highlighting SEM’s utility in organizational psychology research.

Structural models have also been used to elucidate how personal and work environment factors combine to influence job satisfaction, such as occupational self-efficacy’s direct and indirect effects on job satisfaction through learning potential [21].

### 2.5. Limitations of Previous Studies

Despite growing evidence that both personality traits and psychological safety are associated with workplace attitudes, few studies have examined these factors within a single integrated analytical framework in relation to job satisfaction among frontline caregivers. As a result, it remains unclear whether personality traits are directly associated with job satisfaction or whether their associations operate statistically through perceptions of psychological safety.

Addressing this gap is particularly important in care facilities, where work is highly relational and team-based, and where both individual dispositions and organizational climate may jointly shape job satisfaction.

To clarify these relationships, the present study employs a structural modeling approach to examine the associations among personality traits, psychological safety, and job satisfaction, and to test whether psychological safety statistically mediates the association between personality traits and job satisfaction among frontline caregivers.

## 3. Data Collection

This section describes the overview and structure of the dataset used in this study. It also explains the survey procedures, respondent characteristics, and the criteria for selecting the analytical sample.

### 3.1. Problem Statement

To address this research gap, this study examines the relationships among personality traits, psychological safety, and job satisfaction. Let *Y* denote job satisfaction, *M* denote psychological safety, and $X=(X_{1},X_{2},X_{3},X_{4},X_{5})$ denote personality traits, where $X_{1}$ represents extraversion, $X_{2}$ agreeableness, $X_{3}$ conscientiousness, $X_{4}$ neuroticism, and $X_{5}$ openness. In addition, let *Z* denote a vector of control variables, including tenure, age, and facility characteristics.

The relationships among these variables can be expressed by the following structural equations:

(1) $$M=\alpha_{0}+\sum_{i=1}^{5}\alpha_{i}X_{i}+\gamma^{⊤}Z+\varepsilon_{M},$$

(2) $$Y=\beta_{0}+\beta_{M}M+\sum_{i=1}^{5}\beta_{i}X_{i}+\delta^{⊤}Z+\varepsilon_{Y},$$

where $\varepsilon_{M}$ and $\varepsilon_{Y}$ are error terms.

Within this framework, the core research problem is threefold. First, it seeks to determine whether psychological safety exerts a direct effect on job satisfaction ($\beta_{M}\neq 0$). Second, it examines whether personality traits are directly related to job satisfaction ($\beta_{i}\neq 0$). Third, and most importantly, it evaluates whether personality traits are related to job satisfaction indirectly through psychological safety. The indirect (mediated) effect of each personality trait $X_{i}$ on job satisfaction is formally defined as follows:

(3) $$\mathrm{Indirect}\;{\mathrm{Effect}}_{i}=\alpha_{i}\times \beta_{M}.$$

By explicitly modeling these direct and indirect pathways, this study aims to clarify the psychological mechanism through which individual dispositions and workplace environments jointly shape job satisfaction among frontline caregivers. This modeling strategy allows us to examine multiple pathways simultaneously and helps explain why interventions targeting workplace culture and communication may be more effective than approaches focusing solely on individual characteristics.

To examine the practical applicability of the proposed model, it is necessary to capture the key characteristics of all three components through appropriate data collection. Consequently, a survey questionnaire was designed specifically to operationalize the constructs defined in the model. The following sections describe the development process and structural design of the questionnaire used in this study.

### 3.2. Process of Data Collection

Based on the problem statement, we created our own questionnaire for this research. The questionnaire used in this study was developed through a multi-stage review process. First, multiple psychological and job-related scales—such as job satisfaction, emotional labor, fatigue, and work engagement—were comparatively examined. However, it became evident that lengthy questionnaires, including depression and fatigue scales, imposed a substantial response burden and contained items that were not strictly necessary for this study. Therefore, a previously validated seven-item scale for psychological safety was adopted [22], thereby reducing the overall number of items. In the present sample, the internal consistency of the psychological safety scale was acceptable (Cronbach’s α = 0.80).

Job satisfaction was assessed using a single global item reflecting caregivers’ overall evaluation of their work, given the practical constraints of field-based data collection. Previous studies have shown that single-item measures can provide acceptable validity when the construct is conceptualized as a global evaluative attitude rather than a multidimensional set of facets [23]. Moreover, in this study, job satisfaction was treated as an observed outcome variable rather than as a latent construct within the SEM framework, thereby limiting the impact of measurement complexity on model estimation. Also, a concise measure helped reduce response burden and improve data quality among frontline caregivers.

Preliminary testing revealed several issues: respondents found it difficult to understand the intent of some questions, had trouble determining whether the target of the question was the workplace as a whole or specific colleagues, and showed reduced motivation to respond. To address these issues, the present survey added personality as a measurement construct underlying psychological safety. The TIPI-J (10-item version) was adopted as the personality scale [24]. This scale imposes a low response burden, helps maintain respondent motivation, and has been shown in prior research to be associated with workplace behavior and psychological safety.

Given the conceptual proximity among Big Five traits, multicollinearity diagnostics were specifically examined for personality predictors. Variance inflation factors (VIFs) were calculated, as shown in Table 1. All VIF values ranged between 1.17 and 1.57, well below commonly suggested thresholds, indicating no serious multicollinearity concerns.

Based on these considerations, a concise questionnaire structure centered on the following key constructs was adopted in order to minimize response burden while preserving the conceptual structure required for analysis.

In addition, while the first survey obtained responses from a wide range of occupations, including managerial staff, information on work experience—such as length of service and detailed job categories—was insufficient. To address this limitation, the second survey primarily targeted frontline caregivers and additionally collected basic information such as length of service, employment status, and job classification.

Through this process, the questionnaire was developed to simultaneously achieve the following three objectives:

1. Reduction in response burden;
2. Ensuring the measurability of key constructs;
3. Suitability for the operational characteristics of the care industry.

The full questionnaire is shown in Table 2. Personality (TIPI-J) items were rated on a four-point Likert scale ranging from 1 (“does not apply at all”) to 4 (“applies very much”). Unless otherwise noted, all other items were rated on a five-point Likert scale ranging from 1 (“does not apply at all”) to 5 (“applies very much”). It should be noted that in the SEM analyses here, maximum likelihood estimation was applied to the Likert-type responses, which is a common practice in applied organizational research when variables contain four or more response categories. Therefore, the difference in scale range was not considered to substantially influence the estimation of structural relationships.

### 3.3. Result of Data Collection

Two rounds of questionnaire surveys were conducted between 2024 and 2025, yielding a total of 521 responses. The first survey collected 231 responses, with a relatively large proportion from managers or leaders. The survey was distributed via an online platform and LINE. The second survey collected 290 responses, this time mainly from frontline caregivers, but other occupations in care facilities were also involved. At the same time, we updated the questionnaire survey to collect job-related characteristic information, such as years of work at this facility and years of work in the care domain.

From the total of 521 responses collected across two survey waves, the analytical sample was derived based on the following criteria. First, only respondents who identified as frontline caregivers directly engaged in daily care work were included. Manager, administrative personnel, and other non-care roles were excluded. Second, only responses from the second survey wave were retained, as it included complete information on key job-related characteristics such as tenure and employment status. Third, cases with missing data on any of the main variables (personality traits, psychological safety, or job satisfaction) were excluded from the analysis. After applying these inclusion and exclusion criteria, the final analytical sample consisted of 183 frontline caregivers. All analyses involving control variables were conducted using data from the second wave to ensure measurement consistency. Because participation was voluntary and conducted online, self-selection bias cannot be ruled out.

**Figure 1:**
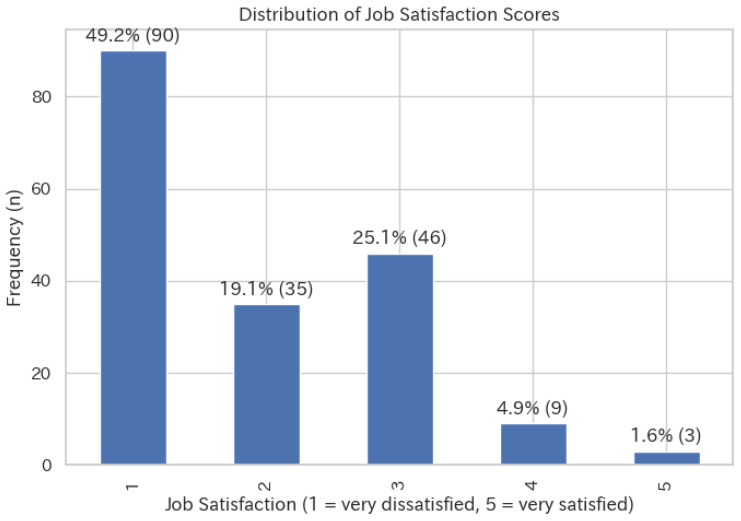
Distribution of job satisfaction scores among frontline caregivers (n = 183). Job satisfaction was measured using a single-item five-point Likert scale (1 = very dissatisfied to 5 = very satisfied). Numbers above the bars represent the frequency of responses in each ordinal category.

We analyzed data for all different positions based on occupation, but the data that met the requirements, including managers, were insufficient for analysis and conclusions. Therefore, the analysis sample ultimately adopted in this study included 183 frontline caregivers from the second survey. Figure 1 is the distribution chart for the job satisfaction score in frontline caregivers. A large proportion of respondents reported very low job satisfaction (score = 1), accounting for the highest frequency in the sample. In contrast, high job satisfaction scores (scores = 4 and 5) were reported by only a small number of caregivers. Overall, job satisfaction in caregivers tends to be generally low, with relatively few respondents expressing high levels of job satisfaction.

## 4. Analysis

**Figure 2:**
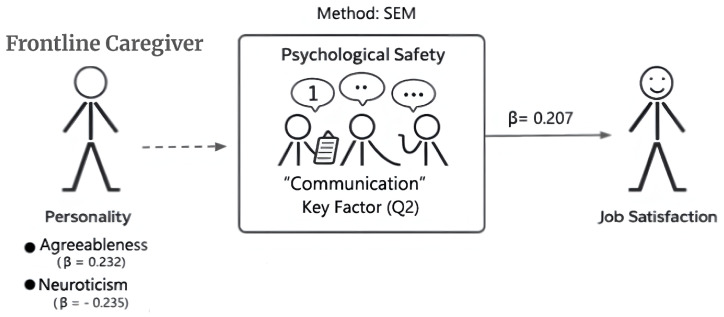
Conceptual framework of this study. The model illustrates the hypothesized relationships among personality traits, psychological safety, and job satisfaction. Standardized coefficients (β) are estimated using structural equation modeling (SEM).

To address three research questions, this study employs multiple analytical methods, each focusing on different parts of the analysis. The conceptual model examined in this study is shown in Figure 2. The model reflects the hypothesized relationships among personality traits, psychological safety, and job satisfaction, which were tested using structural equation modeling (SEM). Only variables consistently measured in the second survey wave were included in the SEM analysis.

First, we used a structural equation model to study the relative importance of personality traits and psychological safety to job satisfaction (RQ1), while evaluating the overall structure of relationships among the three constructs. On this basis, we then conducted a mediation analysis for the parts with less obvious direct effects, discovering that personality traits are associated with job satisfaction mainly through psychological safety (RQ2).

Finally, to better understand how psychological safety plays a role in the care environment, we employed statistical analysis techniques and machine learning models to determine which specific factors of psychological safety most clearly differentiate job satisfaction levels (RQ3) of frontline caregivers.

### 4.1. Personality, Psychological Safety, and Job Satisfaction

In this section, structural equation modeling (SEM) was employed to examine the relationships among personality traits—extraversion, agreeableness, conscientiousness, neuroticism, and openness—psychological safety, and job satisfaction, to clarify their underlying relational structure. The SEM analysis was conducted as a confirmatory test of the hypothesized structural model specified a priori. The mediation structure was tested using bootstrap estimation within this framework. This section describes the details of this analysis. To examine the proposed relationships, we first conducted SEM as the main confirmatory analysis. Item-level comparisons and machine learning models were then used as complementary exploratory approaches to further interpret the findings and assess their practical relevance.

#### 4.1.1. Model Framework

This section aims to clarify how personality and psychological safety are related to job satisfaction and to identify the structural pattern most consistent with the hypothesized model. Job satisfaction is positioned as the outcome, and the directionality of the relationships between personality and psychological safety is examined.

To explore and integrate the directions of influence among multiple variables—such as direct and indirect effects—and the overall relational structure among the three constructs, conventional regression analysis is limited in its ability to capture these simultaneous direct and indirect relationships. Therefore, this study adopts structural equation modeling (SEM), which allows for simultaneous estimation of direct and indirect associations, and seeks to identify the most plausible structure by simultaneously estimating both direct and indirect effects among the three constructs. To assess the statistical significance of indirect effects, bootstrap resampling was applied to estimate confidence intervals for mediation paths. The SEM analysis confirmed that agreeableness was positively associated with psychological safety, whereas neuroticism was indirectly associated with lower job satisfaction via psychological safety. These traits were supported as exerting indirect effects on job satisfaction through the mediation of psychological safety. Multicollinearity diagnostics for personality predictors were examined in the preliminary analyses and indicated no substantial collinearity concerns.

- Effects of personality traits on psychological safety;
- Effects of psychological safety on job satisfaction;
- Direct effects of personality traits on job satisfaction and indirect effects mediated by psychological safety.

#### 4.1.2. Results of Direct Effects

The estimation results of the structural equation modeling (SEM) are presented in Table 3. The model accounted for $R^{2}$ = 0.205 of the variance in psychological safety and $R^{2}$ = 0.152 of the variance in job satisfaction. These values indicate modest but meaningful levels of explained variance, which are typical for cross-sectional organizational research examining complex psychosocial constructs.

Regarding overall model fit, the indices suggested a moderate model fit to the data (CFI = 0.834, TLI = 0.723, RMSEA = 0.081, SRMR = 0.065). Although the CFI and TLI values were below the conventional threshold of 0.90, the RMSEA and SRMR values were within ranges generally considered acceptable in applied organizational research. Given the modest sample size and the inclusion of multiple observed predictors, the overall pattern of fit indices suggests that the model provides a reasonable approximation of the observed covariance structure.

The standardized coefficient β represents the magnitude of the effect, with larger absolute values indicating stronger effects. Statistical significance was determined at a *p*-value of less than 0.05.

(4) PsychologicalSafety→JobSatisfaction

Among personality-related characteristics, agreeableness (β = 0.182, p=0.018) and psychological safety (β = 0.207, p=0.020) showed significant positive effects on job satisfaction. Other personality traits, work-related characteristics, and basic demographic variables such as age did not exhibit significant direct effects.

In contrast, in the paths where psychological safety was specified as the dependent variable, agreeableness (β = 0.232, p=0.006) showed a positive effect, while neuroticism (β = −0.235, p=0.006) showed a negative effect. Other personality traits were not statistically significant.

#### 4.1.3. Results of Indirect Effects

To examine whether psychological safety influences job satisfaction and to test the mediating process (indirect effects), the bootstrap method was used to estimate the confidence intervals and statistical significance of the indirect effects. The bootstrap method is a resampling technique in which a large number of pseudo-samples are generated from the original dataset through sampling with replacement, and statistics are estimated for each sample to assess effect stability and confidence intervals. In particular, for indirect effects and psychometric data based on Likert scales, the distribution of effect sizes often deviates from normality. Because the bootstrap method provides robust estimation under such conditions, it is considered a more appropriate approach.

We conducted 500 resampling iterations and estimated the mean standardized indirect effects and their 95% confidence intervals (CIs). In bootstrap-based mediation analysis, the mean value represents the magnitude of the indirect effect, with larger values indicating stronger indirect influence. The confidence interval reflects the range of uncertainty in which the effect may vary; if this interval does not include zero, the effect can be considered to exist beyond random chance.

Table 4 presents the results of the bootstrap analysis. The following indirect effects were confirmed to be statistically significant:

(5) Agreeableness→PsychologicalSafety→JobSatisfaction

(6) Neuroticism→PsychologicalSafety→JobSatisfaction

These results indicate that agreeableness was indirectly associated with higher job satisfaction through psychological safety within the specified model. The indirect effects of extraversion, conscientiousness, and openness were not statistically significant.

In summary, personality traits were primarily associated with job satisfaction through psychological safety within the tested structural model, and psychological safety emerged as a central variable in explaining variation in job satisfaction.

### 4.2. Psychological Safety and Job Satisfaction

The analyses conducted thus far confirmed through SEM that the direct effects of personality on job satisfaction are limited, and that most influences occur indirectly through psychological safety. In other words, the results suggest that psychological safety may be the central factor influencing job satisfaction. Based on these findings, this section aims to examine the relationship between psychological safety and job satisfaction from a more multifaceted perspective by conducting additional analyses using multiple statistical methods and machine learning models.

#### 4.2.1. Analytical Method

Psychological safety items were treated as scores (mean or sum) in the ANOVA and Tukey analyses, while in some of the machine learning models, the first principal component extracted via principal component analysis (PCA) was used as a feature. The analytical methods employed are as follows:

- ANOVA (Analysis of Variance): Tested whether differences in psychological safety indicators existed among job satisfaction groups.
- Tukey’s Multiple Comparison Test: For psychological safety items that showed significant differences in ANOVA, identified which specific group pairs exhibited significant differences.
- XGBoost: Built a model to predict job satisfaction using a feature set that included basic characteristics such as personality traits, length of service, and facility attributes, as well as the principal component of psychological safety. Feature importance was calculated to evaluate the relative importance of psychological safety.

By combining these multiple methods, the study aimed to examine the impact of psychological safety on job satisfaction from both statistical and machine learning perspectives and to confirm the consistency of the results. The following analyses were exploratory in nature and aimed to further interpret and contextualize the SEM findings at the item level. Across the different analyses, psychological safety was treated as the same underlying construct. In the SEM analysis, it was modeled as a latent factor based on the seven items. In the exploratory analyses, individual items and the first principal component were used to examine different aspects of the same construct from complementary perspectives.

#### 4.2.2. Results of ANOVA (Analysis of Variance)

Comparisons across five job satisfaction groups were conducted for each psychological safety item (Q1–Q7). Significant between-group differences were identified for three items: Q2, Q5, and Q7. For Q2 (p=0.004), Q5 (p=0.041), and Q7 (p=0.011), lower job satisfaction groups tended to report lower psychological safety scores.

After applying a Bonferroni correction for the seven psychological safety items, only Q2 (p=0.004) remained statistically significant. Q5 (p=0.041) and Q7 (p=0.011) did not meet the adjusted significance threshold and were thus deemed non-significant. These results suggest that, among the psychological safety items, the element corresponding to Q2 is most strongly associated with between-group differences in job satisfaction.

#### 4.2.3. Results of Tukey’s HSD (Post-Hoc Multiple Comparisons)

Post-hoc comparisons were conducted for items that showed significance in the ANOVA. Significant differences were observed for the combinations shown in Table 5. Statistically significant differences were confirmed for several group pairs across Q1, Q2, Q5, and Q7. In particular, the lowest satisfaction group (Group 1) showed lower scores than other groups on multiple items. This finding suggests that some aspects of psychological safety may reflect differences in job satisfaction.

#### 4.2.4. Results of XGBoost

To explore whether the observed associations remained evident under nonlinear modeling assumptions, a machine learning approach (XGBoost) was applied as an exploratory analysis. Psychological safety consists of seven items, among which a certain degree of inter-item correlation exists. Therefore, a composite score calculated by a simple average may cancel out overlapping information across items and fail to adequately capture the structural characteristics of psychological safety. To represent psychological safety more comprehensively and robustly, principal component analysis (PCA) was conducted on the seven items prior to building the machine learning model. The first principal component extracted by PCA integrates the shared variance among items and serves as a latent factor that more appropriately represents the overall tendency of psychological safety.

Using a feature set that included the first principal component of psychological safety, an XGBoost model was constructed to predict job satisfaction. Although the predictive performance of the model was limited (RMSE=1.338, $R^{2}=-0.78$), the principal component derived from psychological safety was still ranked as a highly important feature. This indicates that the influence of psychological safety was also confirmed within a nonlinear modeling framework.

#### 4.2.5. Summary of Analysis Results

The results suggest that psychological safety cannot be fully captured by a single factor and instead has a multilayered structure in which different subcomponents play distinct roles. Item-level analyses using ANOVA and Tukey’s HSD revealed significant differences between job satisfaction groups for certain psychological safety items, particularly those centered on Q2.

Furthermore, in the XGBoost analysis, the first principal component, extracted as a common factor underlying the psychological safety item, ranked among the most important features. This finding further supports that psychological safety functions as a central explanatory factor for job satisfaction even in nonlinear models. However, the negative $R^{2}$ indicates limited predictive performance, and therefore, the machine learning results should be interpreted as exploratory rather than confirmatory. Taken together, the confirmatory SEM analysis supported the hypothesized mediation structure, while exploratory item-level and machine learning analyses provided complementary evidence regarding the practical salience of specific psychological safety components. The exploratory findings should therefore be interpreted as supplementary rather than confirmatory.

## 5. Discussion

This study examines how personality traits and psychological safety influence job satisfaction among frontline caregivers, including the presence of mediating mechanisms and the importance of different dimensions of psychological safety. Descriptive analyses suggested that frontline caregivers tended to report relatively lower levels of job satisfaction, underscoring the practical importance of examining workplace factors associated with improvement. Although the CFI and TLI indicate a moderate level of model fit, Kline [25] cautions against relying solely on conventional cutoff values. Therefore, in the present study, model evaluation is based not only on global indices but also on theoretical coherence and the plausibility of the structural relationships. From XGBoost, the negative $R^{2}$ further indicates the limited predictive capacity of the model, reinforcing that the machine learning results should be interpreted strictly as exploratory pattern detection rather than predictive validation. The findings suggest a pattern in which individual characteristics and psychological safety within the work environment were statistically associated with job satisfaction among frontline caregivers in care facilities under the specified structural model.

### 5.1. Contribution

This section outlines the contributions and significance of the present study. The discussion is organized around three research questions, which are systematically addressed and summarized in turn.

RQ1 examined whether personality traits or psychological safety play a more central role in shaping job satisfaction among frontline caregivers. The results consistently indicate that psychological safety is a stronger determinant of job satisfaction than individual attributes such as personality traits, tenure, age, or facility type. Across multiple analytical approaches, psychological safety showed a stable and substantive association with job satisfaction, whereas background factors exhibited only limited explanatory power. This pattern may reflect the highly relational and emotionally demanding nature of frontline caregivers. In such settings, daily tasks are closely intertwined with teamwork, communication, and emotional coordination, making the psychosocial quality of the work environment particularly salient. As a result, job satisfaction appears to depend less on who caregivers are and more on whether they perceive their workplace as psychologically safe. Prior research on nurses, who represent a distinct but similarly high emotional labor profession, also indicates that job satisfaction depends more on perceived psychological safety than on emotional stress pressure [26]. While the present study focuses on frontline caregivers rather than nurses, the shared characteristics of emotional demands and relational work highlight the broader relevance of psychological safety in care settings.

RQ2 focuses on whether psychological safety mediates the relationship between personality traits and job satisfaction. Findings support the statistically supported mediation pattern within the specified model: personality traits indirectly influence job satisfaction through psychological safety, while direct effects remain inconclusive. Specifically, agreeableness positively correlates with psychological safety, whereas neuroticism negatively correlates with it. Both traits were associated with job satisfaction solely through psychological safety.

Although prior research evidence suggests that personality traits are important predictors of job satisfaction in general work settings [27], the present findings show a different pattern among frontline caregivers, in that personality traits were associated with differences in how frontline caregivers perceive and engage with interpersonal work environments. In care facility settings where collaboration and emotional regulation are critical, such perceptions may serve as a direct psychological link between individual traits and work-related outcomes. This mediating perspective helps explain why direct associations between personality traits and job satisfaction are often weak or inconsistent in care-related research.

RQ3 examined which components of psychological safety most strongly explain differences in job satisfaction. Among the psychological safety items, the ease of communication among team members emerged as the most salient factor and remained significant even after correction for multiple comparisons. Research in healthcare settings suggests that hesitation in speaking up and communicating can increase risks for those being cared for [28], and communication is a discretionary behavior shaped by employees’ assessments of interpersonal risk and anticipated reactions from others [29,30]. Taken together, these findings indicate that psychological safety is not a uniform construct; rather, specific components—particularly those related to communication—play a disproportionately important role in shaping job satisfaction in care facilities.

In frontline caregivers, small concerns, such as subtle changes in elderly conditions, near-miss incidents, or accidents in daily life, often need to be communicated promptly to ensure care quality and safety. The present findings suggest that psychological safety was statistically associated with caregivers’ perceived ability to voice concerns without negative consequences.

From this perspective, communication appears to represent a central component within the statistical associations observed between team climate and job satisfaction. This point is also consistent with prior findings in earlier research [31].

Beyond the specific findings of this study, the results may also be interpreted within broader occupational well-being and stress–resource frameworks, such as the Job Demands–Resources (JD-R) model [32], which emphasizes the dynamic balance between work demands and contextual resources in shaping employee well-being. Caregiving professions are widely recognized as emotionally demanding and vulnerable to burnout [33]. Within such contexts, psychological safety may be interpreted, within established theoretical frameworks, as a contextual resource associated with how caregivers experience daily work stressors. The present findings are consistent with this theoretical perspective, suggesting that perceptions of a safe interpersonal climate are linked to higher reported job satisfaction in this care facility context.

When caregivers feel safe to speak up, ask for help, and share concerns, their daily work may feel less overwhelming and more manageable. In this way, psychological safety may help create a work environment where tasks feel clearer and less isolating. From this perspective, personality traits may not directly determine job satisfaction. Rather, they may influence how individuals experience team interactions and whether they feel comfortable seeking support. Overall, the findings suggest that job satisfaction is linked to the interaction between individual characteristics and the psychosocial work environment [34]. Because employee well-being has been associated with care quality and organizational stability [35], improving psychological safety may also be relevant in long-term care settings. Previous intervention studies in long-term dementia care have similarly reported that strengthening communication and team practices is associated with lower perceived stress and better staff well-being [36]. Although the present study did not examine specific interventions, our findings are consistent with this broader line of research, suggesting that supportive team environments play an important role in caregivers’ work experiences.

Although the present study focuses on frontline caregivers rather than nurses, it is important to distinguish between these professional groups. Both roles involve sustained emotional labor, close interpersonal coordination, and responsibility for vulnerable populations, which may explain why psychological safety appears salient in both contexts. However, caregivers in long-term residential care facilities often operate within different organizational structures, professional hierarchies, and resource constraints compared to hospital-based nursing settings. Therefore, while prior findings in nursing research provide a useful reference point, the patterns observed in care facilities should not be assumed to be identical.

These findings should be interpreted within the specific organizational and relational dynamics of long-term residential care facilities for older adults in Japan. Frontline caregivers in such settings typically operate within team-based structures characterized by high interdependence, ongoing interaction with vulnerable older adults, and relatively constrained staffing resources. These contextual features may amplify the salience of interpersonal climate variables such as psychological safety. Therefore, the present findings should not be generalized to hospital-based nursing units or other healthcare systems without further empirical verification.

### 5.2. Limitations and Future Work

This study has several limitations that should be acknowledged. First, although structural equation modeling was used to estimate directional relationships, causal relationships cannot be conclusively established. Future research employing longitudinal designs or intervention-based approaches is needed to clarify the temporal dynamics among psychological safety, personality traits, and job satisfaction. Second, job satisfaction was assessed using a single-item measure, which does not capture its potentially multidimensional nature. Prior research suggests that job satisfaction may consist of distinct facets that could relate differently to workplace conditions [37]. Future studies could therefore incorporate multidimensional job satisfaction scales to examine these domain-specific relationships more closely. In addition, several organizational factors that may influence job satisfaction—such as leadership style, staffing levels, and workload—were not directly measured in the present survey. Including these structural workplace variables would provide a more comprehensive understanding of contextual influences.

In addition, psychological safety was assessed through self-reported perceptions, which may be influenced by individual response tendencies or situational factors. Future studies could incorporate observational or behavioral indicators to further strengthen construct validity. Also, because the final analytical sample was derived from the second survey wave and participation was voluntary, selection bias cannot be excluded. Caregivers who chose to respond may systematically differ from non-respondents in unobserved ways, potentially influencing the observed associations. While the survey included care facilities from various regions of Japan, the sampling approach does not ensure full national representativeness. Therefore, the findings are most appropriately interpreted within comparable care settings. Moreover, although multiple occupational roles participated in the survey, the present analysis focused specifically on frontline caregivers due to limited sample sizes in other categories. Variations in facility type were also not evenly distributed, which may have shaped the observed associations.

Furthermore, the relatively modest sample size and the specific cultural context of Japan may limit the generalizability of the present findings to other healthcare systems. Organizational structures, communication norms, and workplace cultures vary across countries. At the same time, the work conditions of frontline caregivers in long-term residential care facilities differ in important ways from other healthcare settings. Although this contextual specificity constrains generalizability, it also offers value by illustrating how psychological safety operates within a distinct and relatively understudied care environment. By focusing on this setting, the study provides context-grounded evidence that can inform future cross-national comparisons. Future research should replicate the present model with larger, more diverse samples to assess the robustness of the observed associations across cultural contexts.

## 6. Conclusions

This study examined the statistical associations among personality traits, psychological safety, and job satisfaction among frontline caregivers in care facilities. Using multiple analytical approaches, the findings indicate that job satisfaction was more strongly associated with caregivers’ perceptions of psychological safety than with individual personality traits. Although agreeableness and neuroticism were associated with job satisfaction, these associations were primarily observed through their relationships with psychological safety within the specified structural model.

These results suggest that, within care facility contexts, caregivers’ perceptions of being able to communicate openly and express concerns without fear may be closely linked to their reported job satisfaction. Rather than implying causal effects, the present findings highlight the importance of considering both individual characteristics and psychosocial workplace conditions when examining job satisfaction in frontline caregiving roles.

A key contribution of this study lies in its model-consistent evidence that psychological safety is centrally positioned within the pattern of associations observed in care settings. However, given the cross-sectional design and reliance on self-reported measures, these findings should be interpreted as reflecting statistical relationships rather than causal mechanisms.

Future research may benefit from longitudinal or mixed-method designs to further examine how communication processes and psychological safety are experienced in daily care work. Qualitative approaches, such as interviews or observational studies, could provide deeper insight into how caregivers interpret interpersonal dynamics and how these perceptions relate to job satisfaction over time. In addition, incorporating organizational variables such as leadership practices and workload conditions would allow for a more comprehensive understanding of workplace influences in care facilities.

## Figures and Tables

**Table 1:** Variance inflation factors (VIF) for predictor variables.

Variable	VIF
Extraversion	1.58
Agreeableness	1.19
Conscientiousness	1.69
Neuroticism	1.35
Openness	1.21
Psychological Safety	1.37

**Table 2:** Survey on workplace factors influencing job satisfaction of caregivers (all items).

No.	Item	Scale
**A. Personality Traits (TIPI-J/Big Five, 4-point scale)**		
1	I see myself as extraverted, enthusiastic.	1–4
2	I see myself as critical, quarrelsome. (R)	1–4
3	I see myself as dependable, self-disciplined.	1–4
4	I see myself as anxious, easily upset.	1–4
5	I see myself as open to new experiences, complex.	1–4
6	I see myself as reserved, quiet. (R)	1–4
7	I see myself as sympathetic, warm.	1–4
8	I see myself as disorganized, careless. (R)	1–4
9	I see myself as calm, emotionally stable. (R)	1–4
10	I see myself as conventional, uncreative. (R)	1–4
**B. Job Satisfaction and Work Attitudes (5-point scale)**		
11	I feel a strong sense of meaning and value in my current job.	1–5
12	The workload of my current job is too heavy. (R)	1–5
13	I would recommend my current workplace to close friends.	1–5
14	I would like to continue working at this workplace in the future.	1–5
**C. Psychological Safety (7 items, 5-point scale)**		
15	If I make a mistake at work, I am likely to be criticized by members of my workplace. (R)	1–5
16	Members of my workplace can safely point out problems to one another.	1–5
17	Members of my workplace tend to reject people who are different from them. (R)	1–5
18	I have a relationship with my coworkers in which jokes are understood and accepted.	1–5
19	When I am in trouble, I can ask members of my workplace for help.	1–5
20	There are coworkers who intentionally blame or target me. (R)	1–5
21	I am respected at my workplace, and my skills and abilities are valued.	1–5
**D. Background Information (Categorical)**		
22	Please indicate your job position.	Categorical
23	Please indicate your gender.	Categorical
24	Please indicate your age.	Categorical
25	Please indicate your total years of experience in the care industry.	Categorical
26	Please indicate your years of service at your current facility.	Categorical
27	Please describe any positive experiences related to Items 15–21.	Open-ended
28	Please describe any negative experiences related to Items 15–21.	Open-ended
29	Additional comments (if any).	Open-ended

**Table 3:** Estimated structural path coefficients in the SEM.

Dependent Variable	Predictor	Unstandardized Coefficient	Standardized Coefficient	*p*-Value
Job Satisfaction	Agreeableness	0.188	0.182	0.018
Job Satisfaction	Psychological Safety	0.309	0.207	0.020
Job Satisfaction	Years of Experience	−0.126	−0.122	0.088
Job Satisfaction	Gender	−0.278	−0.111	0.117
Job Satisfaction	Conscientiousness	−0.114	−0.111	0.213
Job Satisfaction	Age	−0.085	−0.082	0.265
Job Satisfaction	Openness	0.082	0.079	0.292
Job Satisfaction	Extraversion	0.088	0.085	0.315
Job Satisfaction	Neuroticism	−0.041	−0.040	0.613
Psychological Safety	Agreeableness	0.161	0.232	0.006
Psychological Safety	Neuroticism	−0.163	−0.235	0.006
Psychological Safety	Extraversion	0.086	0.124	0.167
Psychological Safety	Conscientiousness	0.060	0.087	0.348
Psychological Safety	Openness	−0.012	−0.017	0.830

**Table 4:** Indirect effects were estimated as standardized coefficients using bootstrap resampling. The mean values represent the effect sizes of the mediated pathways. Mediation effects were considered significant when the confidence interval did not include zero.

Pathway	Mean	2.5% CI (Lower)	97.5% CI (Upper)
Extraversion → Psychological Safety → Job Satisfaction	0.026	−0.012	0.088
Agreeableness → Psychological Safety → Job Satisfaction	0.050	0.004	0.117
Conscientiousness → Psychological Safety → Job Satisfaction	0.018	−0.017	0.066
Neuroticism → Psychological Safety → Job Satisfaction	−0.048	−0.110	−0.004
Openness → Psychological Safety → Job Satisfaction	−0.004	−0.048	0.033

**Table 5:** Group Comparisons of Psychological Safety Items (Q1–Q7) Using Tukey’s HSD Test (Significant Differences Only).

Item	Group 1	Group 2	*p*-Value
Q1	1	3	0.046
Q2	1	4	0.049
Q5	1	3	0.038
Q7	1	5	0.014
Q7	2	5	0.026

## Data Availability

The database used and analyzed during the current study is available from the corresponding author upon reasonable request due to ethical and privacy restrictions involving personal information of participants.
